# Altered stomatal patterning accompanies a trichome dimorphism in a natural population of *Arabidopsis*

**DOI:** 10.1002/pld3.262

**Published:** 2020-09-03

**Authors:** Noriane M. L. Simon, Jiro Sugisaka, Mie N. Honjo, Sverre Aarseth Tunstad, George Tunna, Hiroshi Kudoh, Antony N. Dodd

**Affiliations:** ^1^ School of Biological Sciences University of Bristol Bristol UK; ^2^ Center for Ecological Research Kyoto University Otsu Shiga Japan; ^3^ John Innes Centre Norwich UK

**Keywords:** development, environmental adaptation, herbivory, stomata

## Abstract

Trichomes are large epidermal cells on the surface of leaves that are thought to deter herbivores, yet the presence of trichomes can also negatively impact plant growth and reproduction. Stomatal guard cells and trichomes have shared developmental origins, and experimental manipulation of trichome formation can lead to changes in stomatal density. The influence of trichome formation upon stomatal development in natural populations of plants is currently unknown. Here, we show that a natural population of *Arabidopsis halleri* that includes hairy (trichome‐bearing) and glabrous (no trichomes) morphs has differences in stomatal density that are associated with this trichome dimorphism. We found that glabrous morphs had significantly greater stomatal density and stomatal index than hairy morphs. One interpretation is that this arises from a trade‐off between the proportions of cells that have trichome and guard cell fates during leaf development. The differences in stomatal density between the two morphs might have impacts upon environmental adaptation, in addition to herbivory deterrence caused by trichome development.

## 1 INTRODUCTION

In *Arabidopsis*, trichomes are large epidermal cells that protrude from the surface of the leaves and petioles. Trichomes play important roles in both biotic defenses and abiotic stress tolerance (Dalin, Agren, Bjorkman, Huttunen, & Karkkainen, 2008; Handley, Ekbom, & Ågren, 2005; Levin, 1973; Mauricio & Rausher, 1997; Sato & Kudoh, 2016; Sletvold & Ågren, 2012; Sletvold, Huttunen, Handley, Kärkkäinen, & Ågren, 2010). However, trichome development appears to impose a fitness cost on growth and reproduction (Kawagoe, Shimizu, Kakutani, & Kudoh, 2011; Mauricio, 1998; Sato & Kudoh, 2016; Sletvold & Ågren, 2012; Sletvold et al., 2010). In addition to trichomes, stomatal guard cells represent another specialized cell type that is present on the leaf surface. Trichome initiation occurs prior to stomatal meristemoid development, and the patterning of trichomes and guard cells appears to be linked (Bean, Marks, Hulskamp, Clayton, & Croxdale, 2002; Bird & Gray, 2003; Galdon‐Armero et al., 2018; Glover, 2000; Larkin, Young, Prigge, & Marks, 1996). Therefore, there might be a trade‐off between trichome and stomatal guard cell development during leaf formation (Glover, Perez‐Rodriguez, & Martin, 1998).

We wished to determine whether trichome formation might be associated with changes in stomatal patterning in natural populations of plants. To achieve this, we investigated stomatal patterning in a naturally occurring population of *Arabidopsis halleri* subsp. *gemmifera* that includes trichome‐forming and glabrous morphs (Kawagoe et al., 2011; Sato & Kudoh, 2016). These trichome morph phenotypes are heritable (Sato & Kudoh, 2015, 2017). The glabrous morphs within this population harbor a large transposon‐like insertion within the *GLABRA1* (*GL1*) gene (Kawagoe et al., 2011). *GL1* is also required for trichome formation in *A. thaliana*, with homozygous *gl1* mutants being glabrous (Oppenheimer, Herman, Sivakumaran, Esch, & Marks, 1991). Our experiments provide new insights into the relationship between stomatal and trichome patterning under natural conditions.

## 2 METHODS

### 2.1 Study site and experimental model

**FIGURE 1:**
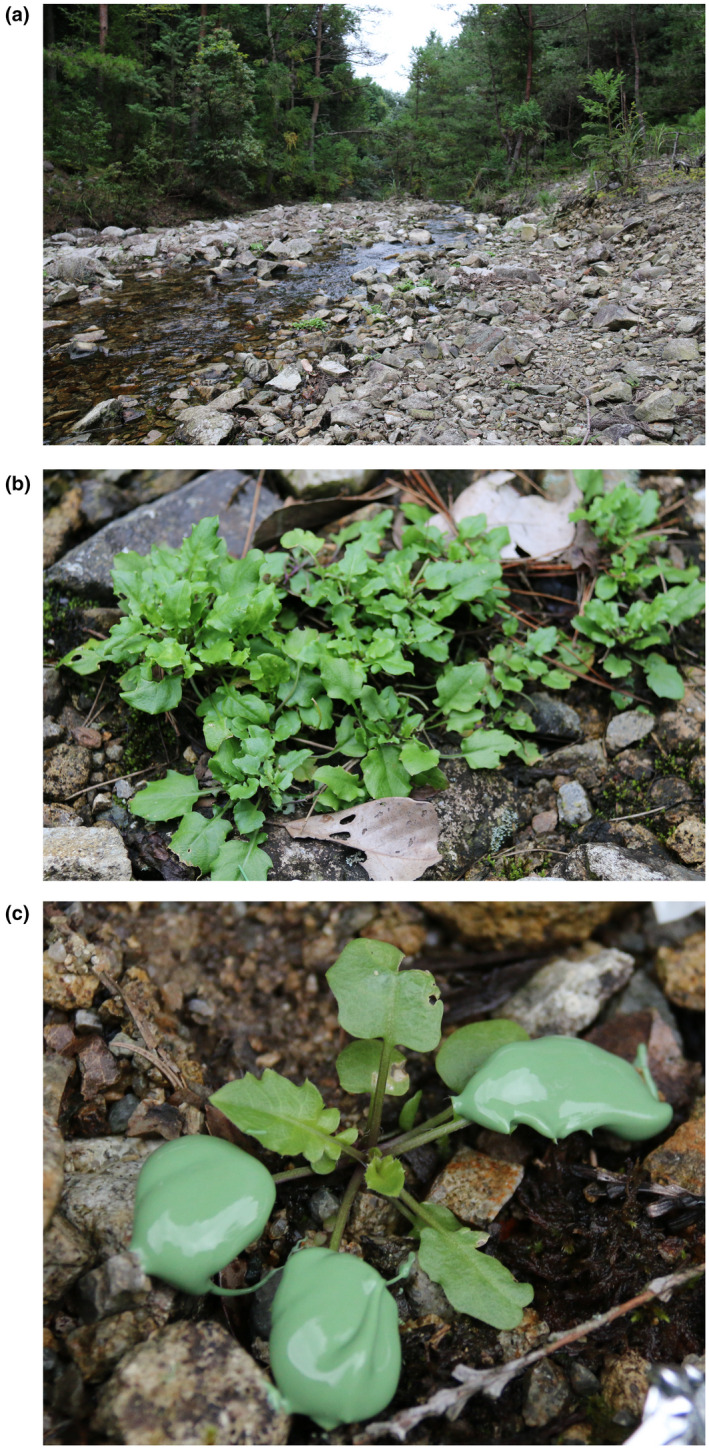
Field sampling of *Arabidopsis halleri* for stomatal density. (a) Overview of field site; (b) Rosette form of *A. halleri* plants when sampling during September 2016; (c) Leaf surface impression acquisition using impression paste. The impression paste is green‐colored and occupies the surface of three rosette leaves

This investigation used a well‐characterized population of *Arabidopsis halleri* subsp. *gemmifera* that is located beside a small stream in central Honshu Island, Japan (Figure 1a) (Omoide‐gawa site, 35°06′ N, 134°56′ E; 230 m altitude) (Aikawa, Kobayashi, Satake, Shimizu, & Kudoh, 2010; Kudoh, Honjo, Nishio, & Sugisaka, 2018). *A. halleri* is metal tolerant and grows essentially as a monoculture at this field site because the water is contaminated by a historical mine (Kudoh et al., 2018). The species was identified by reference to herbarium and museum specimens (Kudoh et al., 2018), and a nearby population that harbors glabrous and hairy morphs supplied material for the genome sequencing and annotation of *A. halleri* (Briskine et al., 2017; Sato & Kudoh, 2017). The only subspecies of *A. halleri* present in Japan is *A. halleri* subsp. *gemmifera* (Honjo & Kudoh, 2019). Sampling occurred during September 2016 (photoperiod approximately 12 hr, with dawn at 05:40 and dusk at 18:10). During this season, *A. halleri* bore larger rosette leaves that are well‐suited for quantification of stomatal density (Figure 1b).

### 2.2 Stomatal density measurement

Eight plants of each trichome morph (hairy or glabrous) were selected at the study site, with individuals chosen such that the replicate plants were distributed evenly across the site. Glabrous and hairy morphs were identified by visual inspection of the leaf surface. It is thought that irradiance and ambient temperature are unlikely to influence the frequency of the morphs (Sato & Kudoh, 2017), but we cannot discount the possibility of microenvironment‐ or field site edge‐effects. Stomatal density was measured by obtaining impressions from the adaxial surfaces of between three and five fully expanded rosette leaves of each plant. We focused on the adaxial surface because this surface also harbors the majority of the trichomes. Between the times of 12:00 and 13:00, President Plus dental impression paste (Coltene) was applied to the adaxial side of each leaf to create a leaf surface impression (Figure 1c). Solidified impression paste was removed from leaves and transported to the laboratory for further processing. First, each impression was assigned a randomly generated number to ensure subsequent steps were performed blind. Each leaf impression was painted with transparent nail varnish (60 s super shine, Rimmel) that, after drying, was peeled away from the dental impression paste using transparent adhesive tape (Scotch Crystal). Next, the adhesive tape was used to attach the nail varnish impression to a 0.8–1 mm thick microscope slide. Leaf impressions were examined using an epifluorescence microscope in white light illumination mode. Images were captured from the center of each leaf half, away from the midrib, using a Hamamatsu camera and Volocity software set to 20x zoom. Two images were captured from each impression, and the number of stomata and pavement cells was counted in an 800 × 800 µm square using the Fiji software to obtain cell density measures. Cell density measures were expressed as per mm^2^ (multiplication by 1.56).

In total, 29 and 31 leaf impressions were obtained in the field from hairy and glabrous plants, respectively. This produced 58 (hairy) and 62 (glabrous) microscopy images for analysis, because two images were captured from each impression. Stomatal index was calculated according to Equation (1). After all measurements, data were disaggregated according to the blinding/randomization scheme. The differences between hairy and glabrous plants were statistically tested by nested analysis of variance, whereby the mean stomatal density or index per replicate plant was nested within the hairy and glabrous morphs. Tests were conducted using the R 3.6.0 software (R Core Team, 2019) and plots generated with the beeswarm R package (v0.2.3) and Inkscape v0.91. No adjustments are applied to photographs in Figure 1.

(1) $$\text{SI}=\frac{s}{s+p}\times 100$$

Derivation of the stomatal index, where SI is the stomatal index, *s* is the number of stomata in the field of view, and *p* is the number of epidermal pavement cells in the field of view.

## 3 RESULTS

We investigated stomatal patterning in naturally occurring hairy and glabrous morphs of *A. halleri* (Sato & Kudoh, 2016). Approximately half of the *A. halleri* population at this study site is glabrous, whilst remaining plants have trichomes (Kawagoe et al., 2011). As trichome initiation occurs prior to stomatal meristemoid formation (Glover, 2000; Larkin et al., 1996), it is likely that trichome and stomatal patterning are linked (Bean et al., 2002), so we hypothesized that this might produce a difference in stomatal density between the two trichome morphs of *A. halleri* under natural conditions.

**FIGURE 2:**
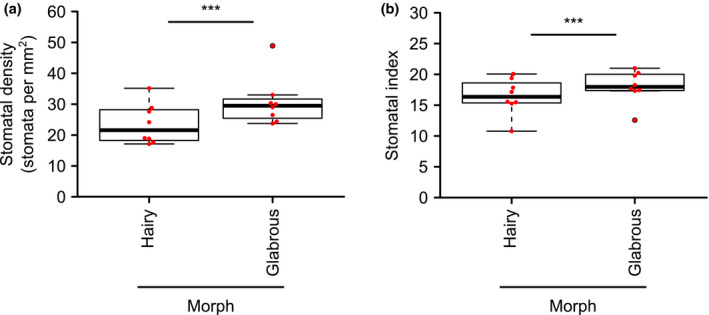
Stomatal density differs between hairy and glabrous morphs within a natural population of *Arabidopsis halleri*. (a) Stomatal density and (b) stomatal index for fully expanded leaves of hairy and glabrous morphs. Each red point represents the mean stomatal density or stomatal index from one individual plant. The mean stomatal density and stomatal index per plant was derived from two microscopy images analyzed from 3 to 4 leaves of each plant. The center line of the boxplot indicates the median. *n* = 8 plants from each morph; analyzed by one‐way nested ANOVA. ***Indicates *p* < .001

We found that the trichome formation dimorphism was accompanied by a difference in stomatal density (Figure 2a; Figure S1; Dataset S1). Fully expanded leaves of glabrous morphs had significantly greater stomatal density on the adaxial surface compared with hairy‐leaved morphs (glabrous: 30.7 ± 2.8 stomata mm^−2^; hairy: 23.6 ± 2.3 stomata mm^−2^; mean ± SEM) (Figure 2a; Table S1; Dataset S1). Furthermore, the stomatal index of the adaxial surface was significantly greater in glabrous morphs (18.04 ± 0.92) compared with hairy morphs (16.44 ± 1.03) (Figure 2b; Table S1). The adaxial surface pavement cell density did not differ significantly between the morphs (Table S1). Although leaf widths varied significantly among plants, they did not differ significantly between the morphs (glabrous: 12.4 ± 0.7 mm; hairy: 13.5 ± 1.0 mm; Figure S2; Dataset S1), suggesting that the stomatal density difference between the morphs is not due to differences in leaf expansion between the morphs (Table S1). Mean stomatal density ranged from 17 to 35 stomata mm^−2^ for hairy morphs and 24–49 stomata mm^−2^ for glabrous morphs (Figure 2a). This stomatal density was lower than for *Arabidopsis thaliana*, which has reported stomatal densities of 180–350 stomata mm^−2^ depending on background accession and growth conditions (Franks, W. Doheny‐Adams, Britton‐Harper, & Gray, 2015; Gray et al., 2000; Zhang, Hu, Cheng, & Huang, 2008). Although lower than in *A. thaliana*, our measurements of stomatal density in *A. halleri* are consistent with a previous report of the stomatal density of A. *halleri* subsp. *gemmifera*, which measured adaxial stomatal density of 46 stomata mm^−2^ at 430 m altitude in autumn rosette leaves, with stomatal density progressively increasing with greater altitudes (Aryal, Shinohara, Honjo, & Kudoh, 2018). Our field site was the lower altitude (230 m), so the lower stomatal densities at our study site (Figure 2a) are congruous with this previous study (Aryal et al., 2018).

## 4 DISCUSSION

Glabrous plants had significantly greater stomatal density and stomatal index compared with hairy plants (Figure 2a,b). As the density of surrounding pavement cells did not vary between the morphs, these differences in stomatal density and index are due to the greater density of stomata in glabrous morphs compared with hairy morphs (Figure 2b). Our field data are consistent with a laboratory‐based study in which transgenic tobacco plants expressing an *Antirrhinum myb*‐like transcription factor, which caused an excess of trichomes, also had significantly reduced stomatal density (Glover et al., 1998). In a segregating tomato population, there is a negative correlation between stomatal and trichome density specifically under drought conditions (Galdon‐Armero et al., 2018). Similarly, the trichome‐bearing Col‐0 accession of *A. thaliana* has lower stomatal density than the glabrous C24 accession (e.g., about 115 mm^−2^ for Col‐0 and 180 mm^−2^ for C24) (Lake & Woodward, 2008; Perazza, Vachon, & Herzog, 1998), although factors other than trichome density are likely to influence stomatal density between the accessions. This suggests that in natural populations of *A. halleri*, there could be a trade‐off between trichome and stomatal development. Since the glabrous *gl1* mutant of *A. thaliana* has a significantly greater density of stomatal units compared with the wild type (Berger, Linstead, Dolan, & Haseloff, 1998) and the glabrous phenotype of *A. halleri* at this study site is associated with an insertion within *GL1* (Kawagoe et al., 2011), it is possible that the *GL1* haplotype influences the stomatal density within this population of *A. halleri*.

In some cases, there does not appear to be a trade‐off between stomatal and trichome density. For example, elevated CO_2_ decreases stomatal density (Woodward & Kelly, 1995), but might also reduce trichome density (Bidart‐Bouzat, Mithen, & Berenbaum, 2005). Therefore, in the future, it could be informative to examine the relationship between stomatal and trichome density under a range of different experimental conditions that apply different types of selection pressure. Furthermore, we sampled the adaxial leaf surface and it is possible that the presence of trichomes might affect stomatal density differently on the abaxial surface because, depending on environmental conditions, abaxial stomatal density of *A. halleri* can be 10%–30% greater than the adaxial surface (Aryal et al., 2018).

Interestingly, trichome production appears to impose a fitness cost. For example, glabrous *A. halleri* plants have 10% greater biomass than hairy plants when grown in the absence of herbivores (Sato & Kudoh, 2016). This cost of herbivore resistance arising from trichome formation also occurs in glabrous and hairy *A. lyrata* (Løe, Toräng, Gaudeul, & Ågren, 2007; Sletvold et al., 2010) and *A. thaliana* (Mauricio, 1998; Mauricio & Rausher, 1997) under experimental conditions excluding herbivores. Whilst this fitness advantage of glabrous over hairy leaves in the absence of herbivory might be due to trichome production (Kawagoe & Kudoh, 2010; Kawagoe et al., 2011; Mauricio, 1998; Mauricio & Rausher, 1997; Sletvold & Ågren, 2012; Sletvold et al., 2010), we suggest that glabrous morphs might also gain an advantage by having a greater density or number of stomata. It has been proposed that increasing the number of stomata could increase carbon assimilation (Lawson & Blatt, 2014). For example, Arabidopsis overexpressing *STOMAGEN* has greater stomatal density and a 30% increase in carbon assimilation compared with the wild type. However, these lines also have higher transpiration rates and consequently lower water use efficiency (Tanaka, Sugano, Shimada, & Hara‐Nishimura, 2013). An alternative interpretation is that differences in the developmental program of the hairy and glabrous morphs might lead to differences in cell or leaf size, which ultimately causes the biomass difference between the morphs. In our samples, the width of fully expanded leaves did not differ significantly between the morphs (Figure S2). Using these leaf width measures as a proxy for leaf size suggests that the biomass difference between the morphs is not due to leaf size differences between the morphs. However, there might be differences in other developmental characteristics that affect biomass, such as leaf thickness, which we did not compare between the morphs.

Optimal stomatal density is important to achieve high photosynthetic rates. A low stomatal density restricts CO_2_ vertical diffusion through the leaf and reduces photosynthetic rates, whilst high‐density stomatal clustering diminishes CO_2_ diffusion and causes low carbon assimilation (Lawson & Blatt, 2014). Both *A. halleri* morphs examined are likely to be within an optimal range of stomatal densities, having evolved and survived under natural conditions. However, the higher stomatal density in the glabrous morph might contribute to its faster growth in the absence of herbivory (Sato & Kudoh, 2016). In the future, it would be interesting to explore this by measuring the CO_2_ assimilation rate of these trichome morphs under laboratory and/or natural conditions. It would also be informative to determine whether the stomatal density difference between the two trichome morphs confers any advantages within microenvironments characterized by differences in water or light availability. The lower stomatal density of *A. halleri* compared with *A. thaliana* (Franks et al., 2015; Gray et al., 2000; Zhang et al., 2008) might reflect differences in growth conditions. An alternative explanation might relate to genome size, because there appears to be a negative correlation between genome size and stomatal density (Beaulieu, Leitch, Patel, Pendharkar, & Knight, 2008), and the genome of *A. halleri* (250 Mb) is approximately double the size of the *A. thaliana* genome (125 Mb) (Briskine et al., 2017; The Arabidopsis Genome, 2000).

In summary, we found that a glabrous morph of *A. halleri* growing under natural conditions had greater stomatal density and stomatal index than a hairy morph. This might contribute to the reported fitness advantage of glabrous plants over hairy plants in the absence of herbivores (Sato & Kudoh, 2017). This differing stomatal density phenotype might derive from the common upstream components in the pathways leading to trichome and guard cell development.

## CONFLICT OF INTERESTS

The authors declare no competing financial interests.

## AUTHOR CONTRIBUTIONS

NMLS, JS, MNH, SAT, GT, HK, and AND performed experimentation and/or analyzed data, and NMLS, MNH, HK, and AND interpreted findings and wrote the paper.

## Supporting information

Dataset S1

Table S1‐Fig S1‐S2

## Data Availability

All data generated during this study are included in the published article and Supplementary Information files.
