# Supplementary material for: Altered stomatal patterning accompanies a trichome dimorphism in a natural population of Arabidopsis
Source: Plant Direct. 2020 Sep 3;4(9):e00262. doi: 10.1002/pld3.262 (PMC7507053; doi:10.1002/pld3.262)
Supplement: Supplementary file 2 — Table S1‐Fig S1‐S2 [file PLD3-4-e00262-s002.pdf]

Table S1. Nested ANOVA analysis of (a) stomatal density, (b) stomatal index, (c) pavement cell density and (d) leaf width. *Df*, degree of freedom;*, **, ***, significant at $p < 0.05$, < 0.01, < 0.001, respectively; NS, not significant at $p >= 0.05$.

(a) Stomatal density

Factor	*Df*	Sum of square	Mean square	*F* value (divided by residuals)	*F* value (divided by plants)
Morph	1	61.1	61.2	19.07***	2.118^{NS}
Plant (nested in morph)	14	404	28.9	9.00***	
Residuals	44	141.1	3.21		

(b) Stomatal index

Factor	*Df*	Sum of square	Mean square	*F* value (divided by residuals)	*F* value (divided by plants)
Morph	1	2177	2177	13.0***	3.994^{NS}
Plant (nested in morph)	14	7631	545.1	3.26**	
Residuals	44	7348	167.0		

(c) Pavement cell density

Factor	*Df*	Sum of square	Mean square	*F* value (divided by residuals)	*F* value (divided by plants)
Morph	1	10070	10070	2.847^{NS}	0.923^{NS}
Plant (nested in morph)	14	152796	10914	3.086**	
Residuals	44	155630	3537		

(d) Leaf width

Factor	*Df*	Sum of square	Mean square	*F* value (divided by residuals)	*F* value (divided by plants)
Morph	1	12.35	12.35	2.77^{NS}	0.59^{NS}
Plant (nested in morph)	14	292.15	20.87	4.68^{***}	
Residuals	44	196.34	4.46		

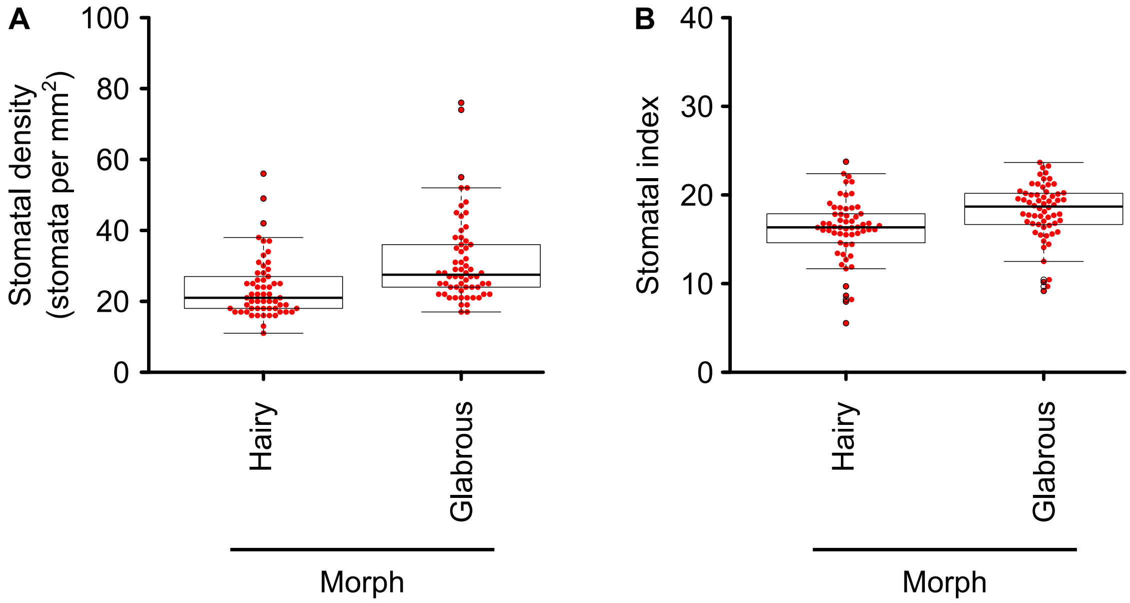

Figure S1. Stomatal density differs between hairy and glabrous morphs within a natural population of *Arabidopsis halleri*. Figure summarizes all data collected for (A) stomatal density and (B) stomatal index of fully expanded leaves of hairy and glabrous morphs. Each red point represents one measurement from one microscopy sample, with 2 samples analyzed from 3-4 leaves from 8 individual plants of each morph. The centre line of the boxplot indicates the median. Data represent a total of 58 and 62 microscopy samples from hairy and glabrous plants, respectively.

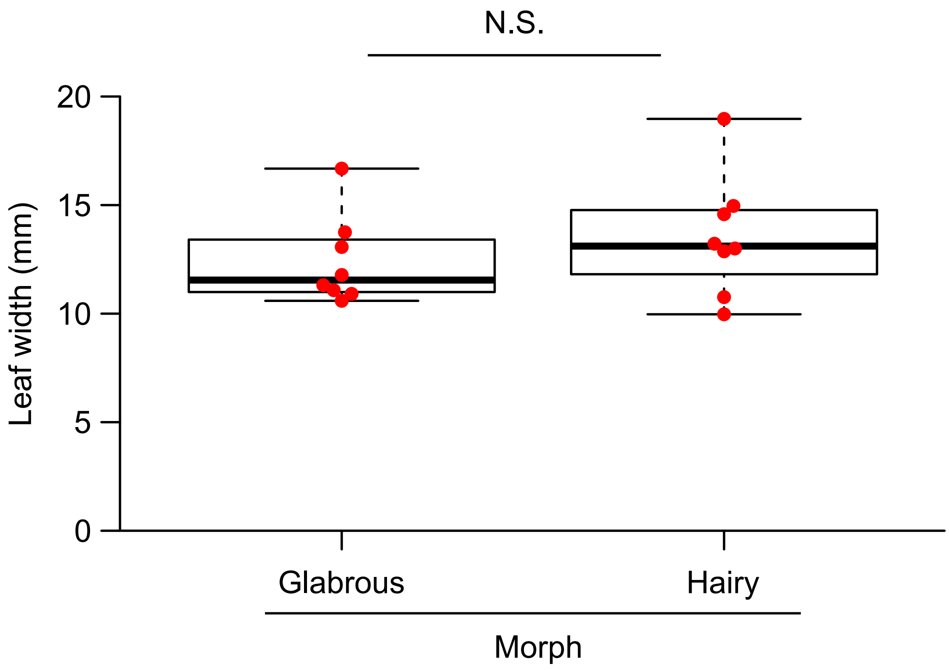

Figure S2. Mean width of fully-expanded leaves does not differ between glabrous and hairy morphs within a natural population of *Arabidopsis halleri*. Each red point represents the mean leaf width from one plant, calculated from the width of either 3 or 4 leaves per plant. 8 replicate plants were sampled for each morph. The centre line of the boxplot indicates the median. Data were analyzed by one-way nested ANOVA, using the mean leaf width per plant as the level of replication.
